# Mapping the Distinctive Populations of Lymphatic Endothelial Cells in Different Zones of Human Lymph Nodes

**DOI:** 10.1371/journal.pone.0094781

**Published:** 2014-04-14

**Authors:** Saem Mul Park, Catherine E. Angel, Julie D. McIntosh, Claudia M. Mansell, Chun-Jen J. Chen, Jonathon Cebon, P. Rod Dunbar

**Affiliations:** 1 School of Biological Sciences, The University of Auckland, Auckland, New Zealand; 2 Maurice Wilkins Centre for Molecular Biodiscovery, The University of Auckland, Auckland, New Zealand; 3 Ludwig Institute for Cancer Research, Austin Health, Heidelberg, Melbourne, Victoria, Australia; University of Sao Paulo–USP, Brazil

## Abstract

The lymphatic sinuses in human lymph nodes (LNs) are crucial to LN function yet their structure remains poorly defined. Much of our current knowledge of lymphatic sinuses derives from rodent models, however human LNs differ substantially in their sinus structure, most notably due to the presence of trabeculae and trabecular lymphatic sinuses that rodent LNs lack. Lymphatic sinuses are bounded and traversed by lymphatic endothelial cells (LECs). A better understanding of LECs in human LNs is likely to improve our understanding of the regulation of cell trafficking within LNs, now an important therapeutic target, as well as disease processes that involve lymphatic sinuses. We therefore sought to map all the LECs within human LNs using multicolor immunofluorescence microscopy to visualize the distribution of a range of putative markers. PROX1 was the only marker that uniquely identified the LECs lining and traversing all the sinuses in human LNs. In contrast, LYVE1 and STAB2 were only expressed by LECs in the paracortical and medullary sinuses in the vast majority of LNs studied, whilst the subcapsular and trabecular sinuses lacked these molecules. These data highlight the existence of at least two distinctive populations of LECs within human LNs. Of the other LEC markers, we confirmed VEGFR3 was not specific for LECs, and CD144 and CD31 stained both LECs and blood vascular endothelial cells (BECs); in contrast, CD59 and CD105 stained BECs but not LECs. We also showed that antigen-presenting cells (APCs) in the sinuses could be clearly distinguished from LECs by their expression of CD169, and their lack of expression of PROX1 and STAB2, or endothelial markers such as CD144. However, both LECs and sinus APCs were stained with DCN46, an antibody commonly used to detect CD209.

## Introduction

The lymphatic sinuses in human lymph nodes (LNs) remain poorly characterized, despite exquisite studies of the lymphatic network in rodent LNs [1]–[3]. Extrapolation from rodent to human LNs is rendered difficult by the obvious differences in the microanatomy, such as the presence of trabeculae and trabecular lymphatic sinuses that radiate from the subcapsular sinus [4]. In addition, several of the markers used to identify lymphatic sinuses in rodent LNs have been difficult to interpret in human LNs as other cell types can also express them. For example blood endothelial cells (BECs) and antigen-presenting cells (APCs) in human tissue have been reported to express the lymphatic vessel endothelial hyaluronan receptor-1 (LYVE1) [4]–[7], routinely used to detect lymphatic sinuses in murine LNs. Other markers once thought to be exclusive for lymphatic endothelial cells (LECs) and BECs, e.g. VEGFR3 and CD144/VE-cadherin respectively, can reportedly be expressed by both endothelial populations in humans [8], [9].

Hence we set out to map a range of markers for LECs in human LNs, and determine which lymphatic sinuses were labeled with each marker. We were also interested in mapping which markers were shared between LECs and sinus APCs. As well as LYVE1, we were interested in better defining which of these cell populations expressed CD209. In our previous work we had characterized a CD209^+^ APC population that occupied many of the lymphatic sinuses, as well as being occasionally present in the paracortex [4]. However, we had noted that the antibody we used to detect CD209 in this earlier study stained more cells in the sinuses than other antibodies to markers expressed by the sinus APCs [4], suggesting this antibody might also stain LECs.

Here we define an exclusive marker that detects all the lymphatic structures composed of LECs in the human LN. For the first time we also describe the distinct distribution of two different populations of LECs forming lymphatic sinuses within human LNs. Finally we establish which markers are capable of readily distinguishing between sinus-type APCs and LECs.

## Materials and Methods

### Human Tissues

We obtained LNs from living donors undergoing surgery and donors post-mortem (Table S1 in File S1). Most LNs studied had no significant histological abnormality, although several had mildly reactive changes. LNs were obtained from the axillary, inguinal, cervical, parotid and mesenteric fields. Patients or the next of kin gave written informed consent, under protocols approved by the Austin Health Human Research Ethics Committee, Heidelberg, Melbourne.

### Multicolor immunofluorescence microscopy

LNs were embedded in TissueTek OCT compound (Sakura Finetek, Zoeterwoude, The Netherlands), snap frozen in liquid nitrogen and sectioned using a cryostat. Sections 5 µm thick were fixed with ice-cold acetone and blocked with 0.25% casein, then incubated with the primary antibodies listed in Table S2 (File S1). The primary antibodies were detected with the corresponding isotype-specific goat anti-mouse or goat anti-rabbit secondary antibodies conjugated to a fluorochrome (Alexa 350, 488, 555 or 647; Invitrogen, CA, USA). The specificity of each secondary antibody was confirmed using an isotype mismatched primary antibody. DAPI was included at 0.0005% w/v with secondary antibody. All data shown is representative of at least 3 or more independent experiments.

The slides were mounted using Prolong Gold (Invitrogen). Sections were visualized with a Leica DMRE fluorescent microscope equipped with the epi-fluorescent filters: UV, 450–490 nm, 530–560 nm and 590–650 nm. Images were acquired at room temperature using 5x/0.15 numerical aperture (NA), 10x/0.30 NA, 20x/0.50 NA and 40x/0.7 NA Leica objectives, a Leica DC500 Digital camera or SPOT camera (Sterling Heights, USA) and analySIS FIVE software (Olympus, Tokyo, Japan). Images were generated using Cytosketch (CytoCode, Auckland, New Zealand) and figures were formatted using Photoshop (Adobe Systems, Mountain View, CA).

## Results

### Mapping lymphatic sinuses in human LNs

To establish a complete map of LECs within human LNs, we stained for nuclear expression of the transcription factor Prospero-related homeobox 1 (PROX1), previously described as a definitive and exclusive marker of LECs in adult tissues [10]. Comparing PROX1 distribution to other endothelial markers allowed us to definitively localize lymphatic channels in relation to blood vessels.

**Figure 1:**
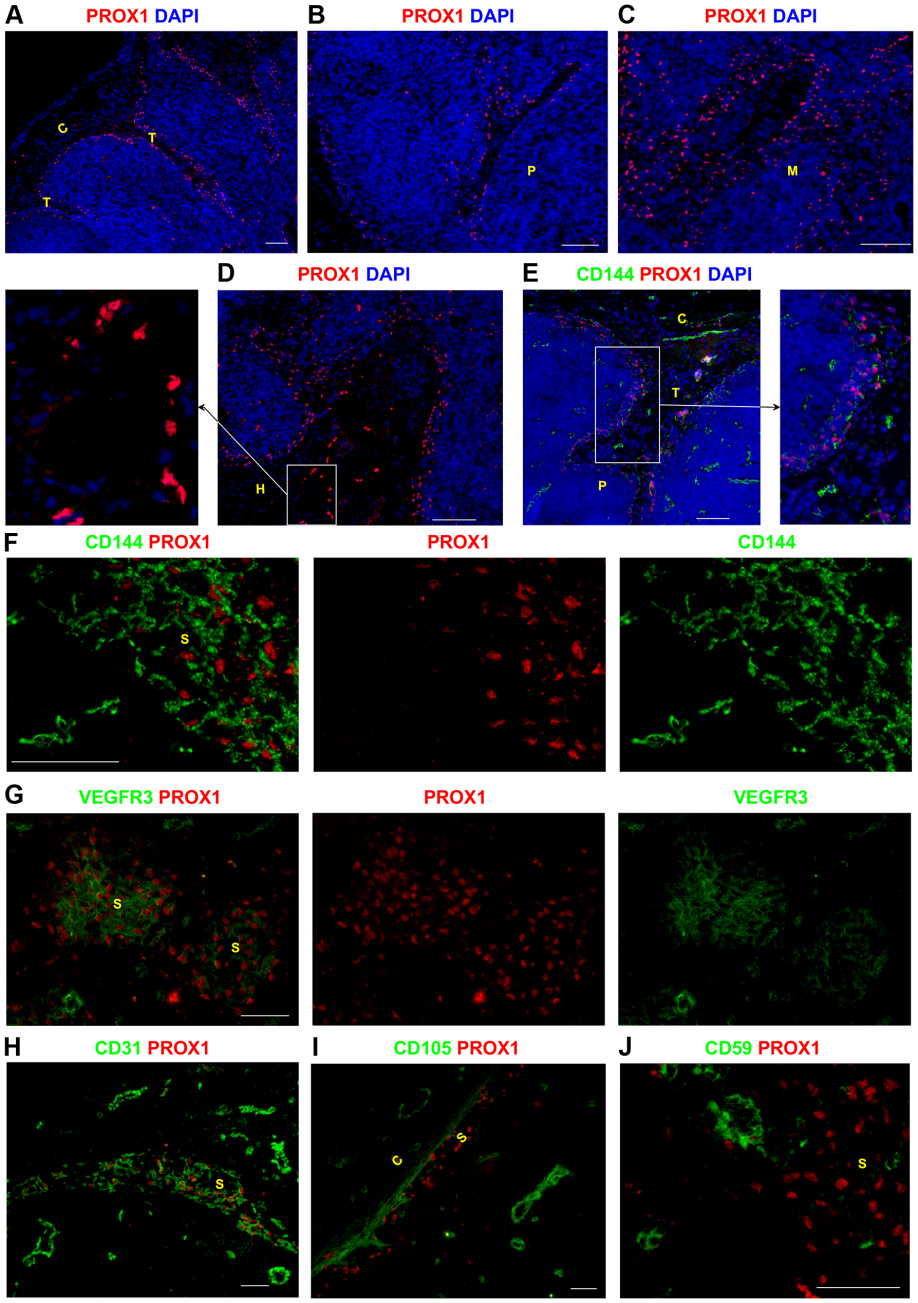
Identifying lymphatic sinuses in the human lymph node using specific markers of lymphatic endothelial cells. Frozen LN sections were probed with an antibody to detect the transcription factor PROX1 to map the lymphatic sinuses in the human LN. The subcapsular and trabecular sinuses were PROX1^+^ (A). PROX1^+^ sinuses were also found in the paracortex (B), the medulla (C), and near the hilum (D). CD144 and VEGFR3 were expressed by lymphatic structures however they were not exclusive LEC markers as they were also expressed by BECs (E–G). Lymphatic sinuses in the medulla expressed varying levels of VEGFR3 (G). Similarly CD31 was also expressed by LECs and BECs throughout the LN (H). LECs lacked expression of CD105 (I) and CD59 (J), whilst these markers were clearly expressed by blood vessels. Blue represents DAPI staining of cell nuclei (A–E). C,capsule; T, trabecula; P, paracortex; M, medulla; H, hilum; S, sinus. Scale bars represent 100 µm (A–E) and 50 µm (F–J).

PROX1 was expressed by the LECs within the sinuses lining the capsule and trabeculae (Figure 1A), the sinuses in the paracortex (Figure 1B) and medulla (Figure 1C), and also the sinus surrounding the hilum (Figure 1D). Ring-like structures composed of PROX1^+^ cells were also detected within the hilum (Figure 1D), indicating the presence of efferent lymphatic vessels (Figure 1D).

PROX1^+^ LECs in the subcapsular, trabecular, paracortical and medullary sinuses co-expressed CD144/VE-cadherin (Figure 1E–F, Figure S1A–D in File S1) in all the LNs examined. PROX1^+^ lymphatic sinuses co-expressed vascular endothelial growth factor receptor 3 (VEGFR3), although there did appear to be some variability in the expression level of this receptor between sinuses, including between sinuses in the same region e.g medulla (Figure 1G). CD31/PECAM-1 was also expressed by PROX1^+^ lymphatic sinuses in the LN (Figure 1H). As reported previously [11], [12], all LECs in the LN expressed podoplanin (Figure S1E in File S1). However, PROX1^+^ sinuses lacked expression of CD105 and CD59 (Figure 1I–J).

In contrast, blood vessels were clearly identified as ring like structures expressing CD144, CD31, CD59 and CD105 but lacking PROX1 (Figure 1E–F, H–J), and were detected throughout the LN. Interestingly, some PROX1^−^VEGFR3^+^ blood vessels were also detected (Figure 1G).

### Two distinctive populations of LECs in different sinuses

**Figure 2:**
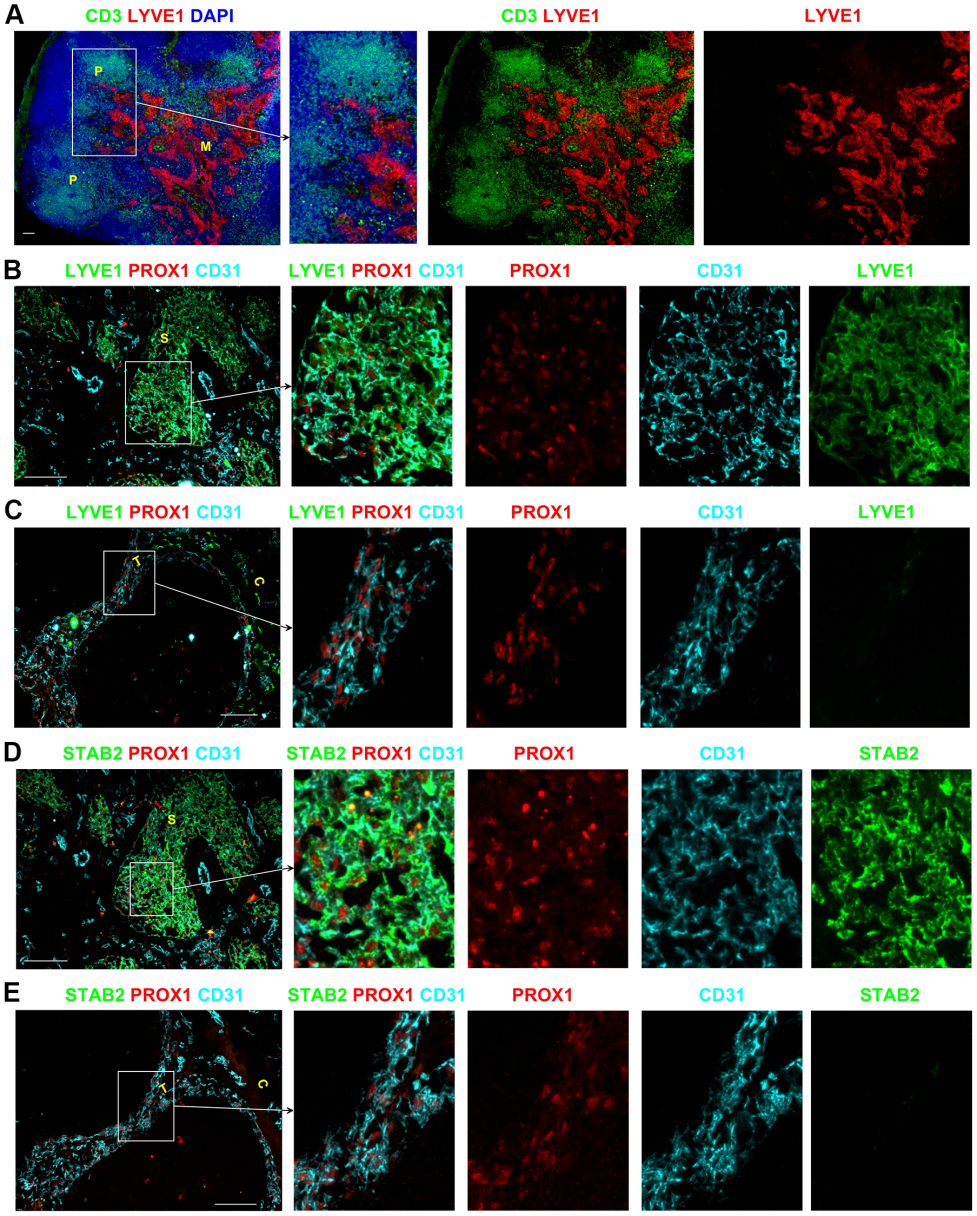
Heterogeneous expression of LYVE1 and STAB2 by endothelial cells in lymphatic sinuses of human LNs. Low magnification images demonstrated that LYVE1 expression is restricted to the paracortical and medullary sinuses whilst the other sinuses in the superficial areas lack expression of this marker (A). PROX1^+^CD31^+^ LECs of the paracortical and medullary sinuses express LYVE1 (B) and STAB2 (D), whereas the LECs in the subcapsular and trabecular sinuses are negative for LYVE1 (C) and STAB2 (E). Blue represents DAPI staining of cell nuclei (A). P, paracortex; M, medulla; C, capsule; T, trabecula; S, sinus. All scale bars represent 100 µm.

Using PROX1 as an exclusive marker of LECs allowed us to distinguish two different subgroups of LECs expressing different levels of LYVE1 and Stabilin-2 (STAB2). In almost all LNs, LYVE1, the LEC marker routinely used in murine LNs, was expressed by the LECs of the paracortical and medullary sinuses (Figure 2A–B, Figure S2A–C in File S1); however LYVE1 was not expressed by the LECs of the subcapsular or trabecular sinuses (Figure 2C, Figure S2D–F in File S1). STAB2 expression was also restricted to the paracortical and medullary sinuses and absent from the subcapsular and trabecular sinuses (Figure 2D–E, Figure S2G–L in File S1). This distinctive expression profile was conserved between the LNs draining peripheral regions (i.e. axillary and cervical) and the mesenteric LNs that drain the gastrointestinal tract and lower abdomen (Figure S2 in File S1). A summary of the phenotype of BECs and LECs in the different sinuses of human LNs is provided in Table 1.

**Table 1:** Contrasting phenotypes of the lymphatic endothelial cells and blood endothelial cells in human lymph nodes.

	PROX1	LYVE1	STAB2	DCN46	VEGFR3	CD144	CD31	CD59	CD105
LECs in subcapsular and trabecular sinuses	+	−	−	−	+ v	+	+	−	−
LECs in paracortical sinuses	+	+/−	+/−	+/−	+ v	+	+	−	−
LECs in medullary sinuses	+	+	+	+	+ v	+	+	−	−
LECs in sinuses surrounding the hilum	+	+	+	+	+ v	+	+	−	−
BECs	−	−	−	−	+ v	+	+	+	+
+: consistent expression by all cells									
+ v: varying level of expression by all cells									
+/−: majority of cells positive									
−: no expression on any cells									
LECs: lymphatic endothelial cells									
BECs: blood endothelial cells									

### Distinguishing between sinus-resident APCs and LECs

We previously used the antibody DCN46 to identify CD209^+^ APCs in human LNs [4]. We had noted that this antibody stained more cells within the sinuses than antibodies to CD206, CD14, or CD68 [4], which also labeled sinus APCs.

**Figure 3:**
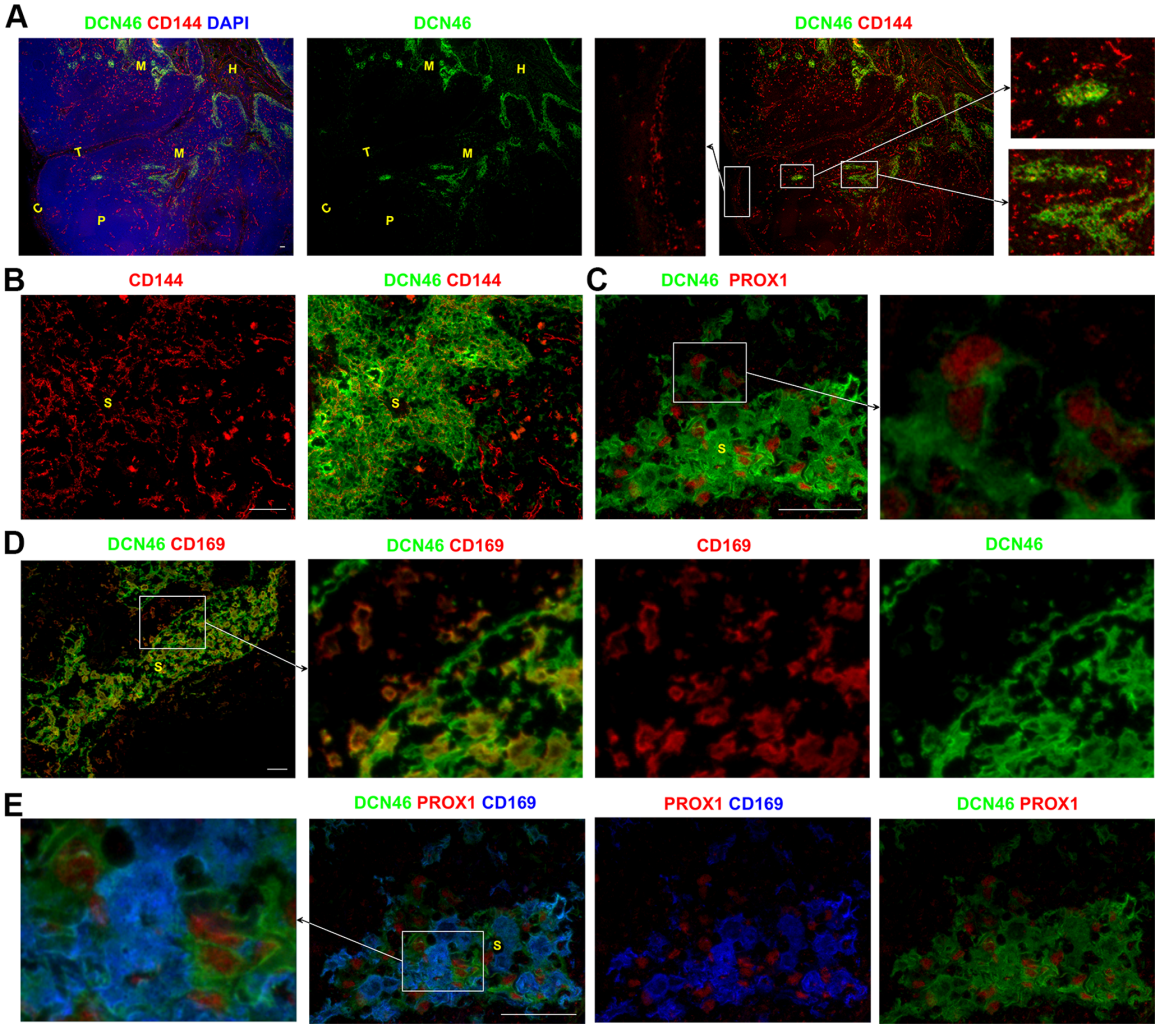
DCN46+ LECs and APCs can be identified by their expression of PROX1 and CD169 respectively. A dense body of cells staining for DCN46 was detected in the paracortical and medullary sinuses, which were located by their distinctive pattern of CD144 expression (A–B). In these regions, subsets of DCN46^+^ cells were found to co-express the exclusive lymphatic endothelial cell transcription factor PROX1 (C) and the APC marker CD169 (D). Simultaneous detection of DCN46, PROX1 and CD169 confirmed that the DCN46^+^ cells in the paracortical and medullary sinuses comprised two distinct populations: DCN46^+^PROX1^+^CD169^−^ LECs and DCN46^+^PROX1^−^CD169^+^ APCs (E). Blue represents DAPI staining of cell nuclei (A). C,capsule; T, trabecula; P, paracortex; M, medulla; H,hilum; S, sinus. All scale bars 50 µm (A–E).

DCN46 stained a dense network of cells in the paracortical and medullary sinuses of human LNs (Figure 3A–B). DCN46 also stained cells in the sinus lining the hilum region (Figure 3A). In contrast, the DCN46 antibody did not recognize the subcapsular or trabecular sinuses in the vast majority of LNs studied (Figure 3A). In the paracortical and medullary sinuses containing the densely packed DCN46^+^ cells, LECs could readily be identified as a CD144^+^ mesh (Figure 3A–B, Figure S3A–C in File S1) with nuclear expression of PROX1 (Figure 3C). Co-staining for PROX1 and DCN46 revealed that a subset of the DCN46^+^ cells in the paracortical and medullary sinuses co-expressed this exclusive LEC marker (Figure 3C), confirming that the DCN46 antibody recognizes LECs.

A proportion of the DCN46^+^ cells in the paracortical and medullary sinuses also expressed the APC marker CD169 (Figure 3D). Simultaneous detection of DCN46, CD169 and PROX1 demonstrated that the DCN46^+^CD169^−^ cells were PROX1^+^ indicating that they represent the LECs (Figure 3E). In contrast the DCN46^+^CD169^+^ cells lacked PROX1 expression confirming that these cells represent the APCs that colonize the sinuses (Figure 3E).

**Figure 4:**
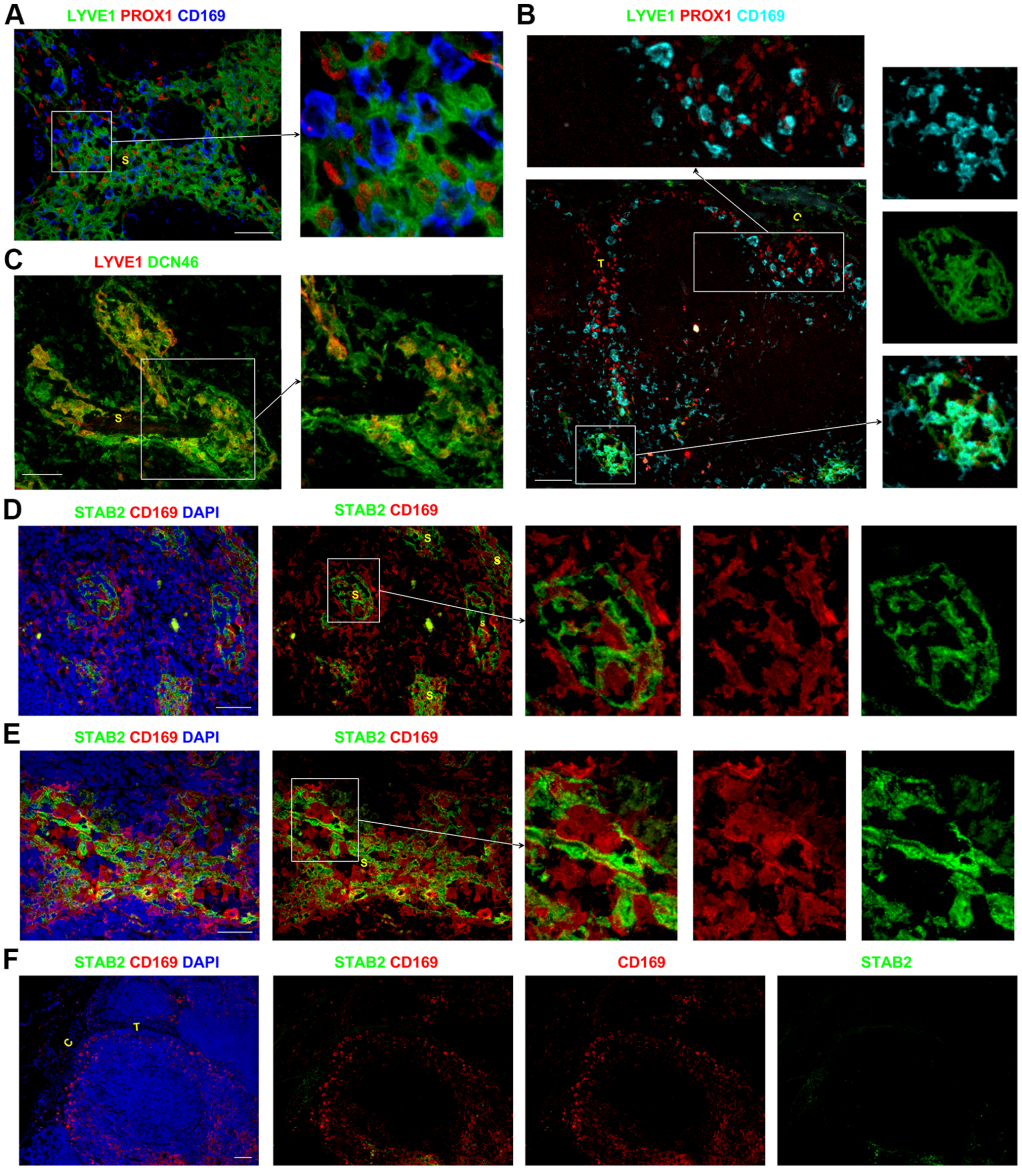
Assessing the phenotype of the APCs and LECs in the lymphatic sinuses. Within the sinuses in the paracortex and medulla, the PROX1^+^CD169^−^ LECs expressed the lymphatic marker LYVE1. The majority of PROX1^−^CD169^+^ APCs in the paracortical and medullary sinuses lacked LYVE1 expression (A), although rare cells in these sinuses appeared to co-express CD169 and LYVE1 (B). CD169^+^ APCs in the subcapsular and trabecular sinuses lacked LYVE1 (B). LYVE1 was expressed by a subset of the DCN46^+^ cells in the medullary sinuses, which are likely to represent the LECs (C). CD169^+^STAB2^−^ APCs were closely associated with the CD169^−^STAB2^+^ LECs in the paracortical and medullary sinuses (D–E). The subcapsular and trabecular sinuses did not express STAB2 (F), although CD169^+^ APCs were present. Blue represents DAPI staining of cell nuclei (D–F). C,capsule; T, trabecula; S, sinus. Scale bars represent 50 µm (A, C–E) and 100 µm (B, F).

LYVE1, although routinely used a LEC marker, has been reported to be expressed by some APCs [4]–[7]. In the paracortical and medullary sinuses, the majority of LYVE1^+^ cells were PROX1^+^ and did not express the sinus APC marker CD169 (Figure 4A). Occasional LYVE1^+^ cells in these sinuses did appear to co-express CD169 (Figure 4B, right panels), although it was difficult to distinguish between closely associated cells. Co-staining for LYVE1 and CD45 showed that the majority of LYVE1^+^ cells in the paracortical and medullary sinuses lacked CD45 (Figure S3D in File S1), suggesting these sinus LYVE1^+^ cells were mostly LECs and not APCs. Interestingly, some LYVE1^+^ cells were found within the capsule above the subcapsular sinus (Figure 2C, 4B, and Figure S3E-F in File S1). These capsular LYVE1^+^ cells expressed CD45 and CD68 consistent with them being APCs, while the LYVE1^+^ LECs in the sinuses were negative for CD45 and CD68 (Figure S3D–G in File S1).

The subcapsular and trabecular sinuses in the majority of LNs studied lacked LYVE1 expression, and CD169^+^ LYVE1^−^APCs could clearly be detected in these sinuses (Figure 4B). Similar to the distribution of LYVE1, STAB2 was also only expressed by the LECs in the paracortical and medullary sinuses, whilst the subcapsular and trabecular sinuses lacked expression of STAB2. CD169^+^ APCs that lacked STAB2 were easily detected in amongst the STAB2^+^ CD169^−^ LECs in the paracortical and medullary sinuses (Figure 4D–E, Figure S3H–J in File S1). CD169^+^ APCs were not restricted to the sinuses, and were also detected scattered throughout the parenchyma (Figure 3D, 4B and D).

Hence DCN46 stains both the APCs and the LECs in the paracortical and medullary sinuses. CD169 exclusively stains the APCs in all sinuses, with no expression of the LEC markers PROX1 and STAB2. It is possible that small numbers of these sinus CD169^+^ APCs may express LYVE1, but this appearance could also be due to the contiguous nature of these CD169^+^ APCs with LYVE1^+^ LECs. Therefore simultaneous co-staining for CD169 and PROX1 is the only combination assessed which will definitively distinguish APCs from LECs in all the sinuses of the human LN. In the paracortical and medullary sinuses, co-staining for CD169 and STAB2 will also clearly delineate these two cell populations (Figure 4D–E, Figure S3H–J in File S1). Furthermore it is notable that DCN46 together with LYVE1 and STAB2 only stain the LECs in the paracortical and medullary sinuses in most LNs examined (although DCN46 also stains APCs). This suggests that LECs in different sinuses may co-regulate expression of LYVE1, STAB2 and the DCN46 antigen.

### CD209 expression by sinus-resident APCs and LECs

**Figure 5:**
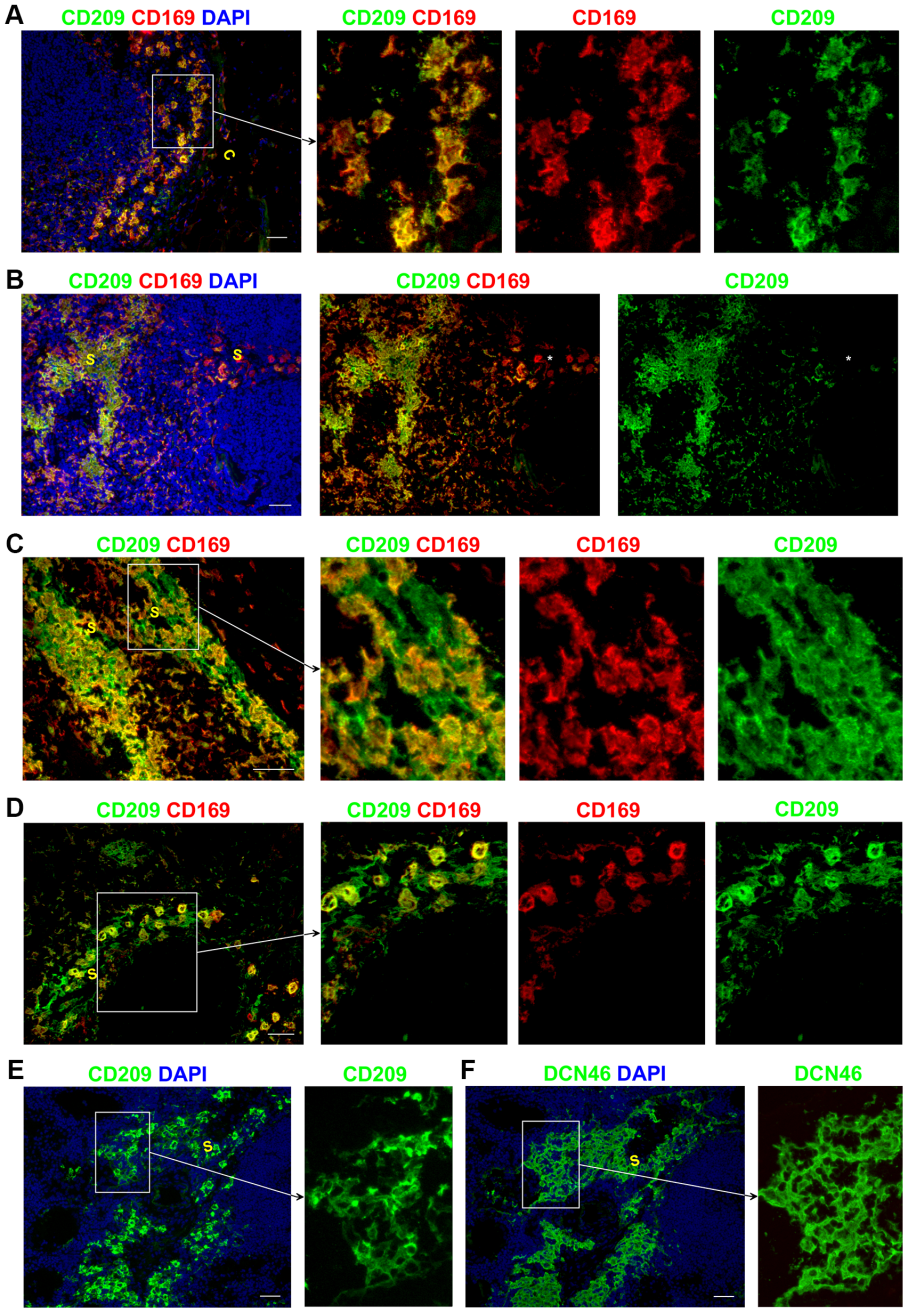
CD209 expression in lymphatic sinuses. CD169^+^ APCs also expressed CD209 (A–D). Rare CD169^+^ CD209^−^ APCs were detected in one of the LNs assessed (B: middle panel marked with asterisk). CD209^+^CD169^−^ cells in the paracortical and medullary sinuses are likely to represent LECs (C–D). Serial LN sections stained with anti-CD209 (E) and DCN46 (F) showed that far fewer cells were stained by anti-CD209 antibody than by DCN46. Blue represents DAPI staining of cell nuclei (A–B, E–F). C,capsule; S, sinus. All scale bars represent 50 µm.

Although it was originally marketed as specific for CD209, the DCN46 antibody has recently been shown to cross-react with CD299/L-SIGN/CLEC4M [13], a molecule previously reported to be expressed by sinusoidal endothelial cells in human LNs and liver [14], [15]. To confirm which of the DCN46^+^ cells in the sinuses were CD209^+^, we used a second antibody to CD209 (Figure 5A–D, Figure S4 in File S1). In all sinuses, the majority of CD169^+^ APCs were CD209^+^ (Figure 5A–D), consistent with the phenotype of the resident APCs we previously reported: CD209^+^ CD206^+^ CD14^+^ CD68^+/−^ [4]. CD169^+^ CD209^−^ APCs were rare in the sinuses. Occasional CD209^+^ CD169^−^ cells with the morphology of LECs were observed interspersed between CD169^+^ CD209^+^ APCs in the paracortical and medullary sinuses (Figure 5C–D); however whether these cells are indeed LECs or APCs remains to be determined. Staining serial sections of human LNs with DCN46 and an antibody specific for CD209, showed that far fewer cells were stained by the anti-CD209 antibody than by DCN46 (Figure 5E–F). Since DCN46 stained more cells than anti-CD209 in the same sinus, we conclude that DCN46 is likely to be recognizing CD299 on LECs in the paracortical and medullary sinuses (but not the subcapsular and trabecular sinuses).

## Discussion

A better understanding of the structures controlling immune cell trafficking within human LNs is likely to lead to new insights into disease processes, and also improve the development of new immunomodulatory therapy. In this paper we characterize the molecular phenotype and distribution of LECs in human LNs.

To improve our ability to visualize the lymphatic sinuses in human LNs, we first conducted a detailed analysis of the lymphatic endothelium in different regions of the human LN using 4-color immunofluorescence microscopy. For the first time in human LNs, we demonstrate that PROX1, a transcription factor regulating lymphatic development [16], is the only exclusive marker currently available for LECs within human LNs, capable of marking lymphatic sinuses and vessels in every zone of the LN. This work is consistent with an earlier study that concluded PROX1 is constitutively expressed by all LECs in human tissues, although this study did not assess LNs [10]. In contrast to PROX1, expression of LYVE1, one of the most widely used markers of LECs in normal and tumor tissues, was restricted to the paracortical and medullary sinuses in most LNs, and was not expressed by PROX1^+^ LECs in the subcapsular and trabecular sinuses. This distribution pattern differs from that observed in murine LNs, where LYVE1 is expressed by sinuses throughout the LN [2], [17]. Previous studies assessing LYVE1 in human LNs did not report the disparity of expression between sinuses in different regions, perhaps due to their focus on medullary sinuses [9], [14]. Interestingly, a study assessing LNs from healthy adult cynomolgus macaques did demonstrate that LYVE1 mRNA expression was restricted to the cortical and medullary regions, whilst was absent from the subcapsular sinus [18]. STAB2, also known as a hyaluronan receptor, was expressed by LECs in similar locations to LYVE1, namely the paracortical and medullary sinuses, but not the subcapsular and trabecular sinuses in most LNs (Table 1). STAB2, like LYVE1, is therefore not a comprehensive marker of LECs in human LNs. Interestingly the LECs that form the paracortical and medullary sinuses also bound the antibody DCN46, originally designed to be specific for CD209 but subsequently revealed to also cross-react with CD299/L-SIGN [13]. In contrast, the majority of sinuses elsewhere in the LN did not bind DCN46. Restricted expression of LYVE1, STAB2 and DCN46 by LECs in the paracortical and medullary sinuses but not by the subcapsular and trabecular sinuses suggested that two different LEC populations occupied different sinuses of the same LN. Alternatively, this feature might indicate two different phenotypic states of the LEC population in response to environment factors, as suggested by an earlier *in vitro* study using cultured LECs [19]. In addition to STAB2 being a hyaluronan receptor, it has been shown to support the integrin-mediated adhesion of lymphocytes to the hepatic sinusoidal endothelium [20]. Furthermore LYVE1 is also postulated to play a role in regulating cell trafficking [21]. Collectively, the current literature suggests that LYVE1 and STAB2 are restricted to certain subsets of endothelial cells, such as sinusoidal endothelial cells in the spleen, where lymphocyte adhesion occurs under unique low shear conditions. It is therefore possible that expression of these particular markers by the LECs in the paracortical and medullary sinuses mediate lymphocyte adhesion to the LECs, thereby facilitating migration. In this context the lack of expression of these molecules by the LECs in the subcapsular and trabecular sinuses is intriguing, and suggests quite different interactions between lymphocytes and LECs in these areas – or perhaps a paucity of lymphocyte migration at these sites.

This picture is underscored by the likelihood that DCN46 expression by LECs in paracortical and medullary sinuses is largely due to CD299/L-SIGN expression. The name L-SIGN signifies expression on both liver and LN sinusoidal endothelium, as demonstrated in an early immunohistochemical study of human LN using a polyclonal antibody raised against a peptide unique to L-SIGN [15]. Co-expression of CD299/L-SIGN with LYVE1 was subsequently documented in a study of human LNs using another polyclonal antibody raised against a similar peptide [14]. The latter study noted that CD209/DC-SIGN and CD299/L-SIGN were expressed on different cell populations that intercalated in the sinuses [14], largely consistent with our findings. However these authors concluded that the CD299/L-SIGN^+^ cells were *near* sinuses rather than being an integral part of the medullary sinus structures, and that T-lymphocytes would need to cross multiple layers of these endothelial cells to enter the sinuses [14]. Our DCN46 stains suggest that the entire mesh of inter-digitating LECs within paracortical and medullary sinuses may express CD299/L-SIGN. Like CD209, CD299/L-SIGN is noted to bind ICAM3 with high affinity [22], consistent with a role in leukocyte adhesion. Recent findings that leukaemic blast cells bind to CD209 and CD299 [23] also raise the possibility that haematological cancer cells may interact preferentially with LECs in the paracortical and medullary sinuses rather than in the other sinuses. Hence these data support the concept that LECs in different sinuses may have quite different interactions with immune cells based on different expression of molecules such as LYVE1, STAB2 and CD299.

We also clarified the expression pattern of another marker often used to identify LECs in human tissue, the receptor for the vascular growth factors C and D, VEGFR3 [8], [9]. Our data confirm that lymphatic sinuses express this receptor in human LNs as well as some blood vessels. An earlier study noted that BECs in malignant tumors can also express VEGFR3 [8], but to our knowledge this has not been previously observed in normal or mildly reactive tissues. CD144 has been used as a BEC marker in the past, however recent studies have indicated that LECs in murine tissues including the LN can also express this adhesion molecule [24], [25]. We confirm that LECs in human LNs also express CD144 and that CD144^+^ LECs in the sinuses form quite a distinctive pattern consistent with its junctional localization, appearing as a mesh like structure spanning all lymphatic sinuses throughout the human LN. Although previous findings also suggested that human LN sinuses express CD144, LECs were not definitively identified using PROX1 [9]. The role of CD144 on LECs remains unclear, although studies of murine LECs in peripheral tissues suggest that CD144 may be involved in regulating lymphatic integrity [26]. Collectively these data demonstrate that many putative LEC markers are also expressed by BECs or are not expressed by all LECs in human LNs from the different locations assessed, and that PROX1 is the only marker screened that is exclusively expressed in all lymphatic sinuses. In this study it was not possible to record the positions in the LN chain of each of the LNs analyzed, although we would expect this to vary.

This work has also extended knowledge of the molecular phenotype of APCs in the sinuses from that we had previously published [4]. CD169 is able to clearly distinguish PROX1^–^ sinus APCs from LECs. CD169 is expressed not only by APCs in the sinuses, but also by APCs with the same molecular phenotype in the parenchyma. CD169^+^ sinus APCs account for the vast majority of the CD209 signal from the sinuses. But we did not find strong evidence that sinus APCs can express LYVE1 as previously reported [5]–[7]: some of our images gave an impression of co-expression, but closer inspection showed this might be due to the close juxtaposition of APCs and LECs within the sinuses. We therefore propose that APCs can be distinguished from LECs in sinuses by their lack of staining for PROX1 and STAB2 as well as their expression of CD169. Collectively our data provide new markers and maps of the lymphatic sinuses in human LNs that will enable new studies of human LNs in health and disease.

## Supporting Information

File S1 **This file contains Figure S1 to S4 and Table S1 and S2.**
(DOC)
