# Correction: Mapping the Distinctive Populations of Lymphatic Endothelial Cells in Different Zones of Human Lymph Nodes

**DOI:** 10.1371/journal.pone.0106814

**Published:** 2014-08-26

**Authors:** The PLOS ONE Staff

The fourth author’s name is spelled incorrectly. The correct name is: Claudia J. Mansell. The correct citation is: Park SM, Angel CE, McIntosh JD, Mansell CJ, Chen C-JJ, et al. (2014) Mapping the Distinctive Populations of Lymphatic Endothelial Cells in Different Zones of Human Lymph Nodes. PLoS ONE 9(4): e94781. doi:10.1371/journal.pone.0094781
