# Supplementary material for: Mapping the Distinctive Populations of Lymphatic Endothelial Cells in Different Zones of Human Lymph Nodes
Source: PLoS One. 2014 Apr 14;9(4):e94781. doi: 10.1371/journal.pone.0094781 (PMC3986404; doi:10.1371/journal.pone.0094781)
Supplement: File S1 — This file contains Figure S1 to S4 and Table S1 and S2. (DOC) [file pone.0094781.s001.doc]

**Table S1. Lymph node samples assessed in this study**

All of the LNs above displayed normal architecture including the paracortex, follicles, medulla, and endothelial and stromal networks.

|  | **Location** | **Features** |
| --- | --- | --- |
| 1 | Inguinal | Mild paracortical expansion, very occasional secondary follicles, lymphadenopathy |
| 2 | Axillary | Very occasional secondary follicles, lymphadenopathy |
| 3 | Axillary | Post mortem, 16hr delay |
| 4 | Axillary | Non-specific reactive features, very occasional secondary follicles |
| 5 | Axillary | Florid reactive follicular hyperplasia without specific diagnostic features |
| 6 | Axillary | Reactive follicular hyperplasia and transformed germinal centres |
| 7 | Cervical | Non-specific reactive features, occasional secondary follicles |
| 8 | Cervical | Florid reactive changes, occasional secondary follicles |
| 9 | Cervical | Reactive hyperplasia with fibrosis, several large secondary follicles |
| 10 | Cervical | Reactive follicular hyperplasia |
| 11 | Parotid | Lymphoid hyperplasia, occasional secondary follicles |
| 12 | Mesenteric | Very occasional secondary follicles |
| 13 | Mesenteric | Occasional secondary follicles |
| 14 | Mesenteric | Very occasional secondary follicles |

**Table S2. List of antibodies used in multicolor immunofluorescence microscopy**

| **Target** | **Clone** | **Manufacturer** |
| --- | --- | --- |
| **Mouse monoclonal antibodies** | | |
| CD31 | WM59 | BD Biosciences |
| CD45 | F10-89-4 | Abcam |
| CD59 | p282 H19 | BD Biosciences |
| CD68 | Y1/82A | Biolegend |
| CD105 | SN6 | Serotec |
| CD144 | 55-7H1 | BD Biosciences |
| CD169 | HSn7D2 | Abcam |
| CD209/CD299 | DCN46 | BD Biosciences |
| CD209 | 9E9A8 | Biolegend |
| LYVE1 | 264712 | R&D |
| PROX1 | 4G10 | Sigma |
| VEGFR3 | 9D9F9 | Millipore |
| **Rat monoclonal antibodies** | | |
| Podoplanin | NC-08 | Biolegend |
| **Rabbit polyclonal antibodies** | | |
| CD3 |  | Zymed |
| LYVE1 |  | Abcam |
| PROX1 |  | Abcam |
| STAB2 |  | Sigma |

**Figure S1.**

**
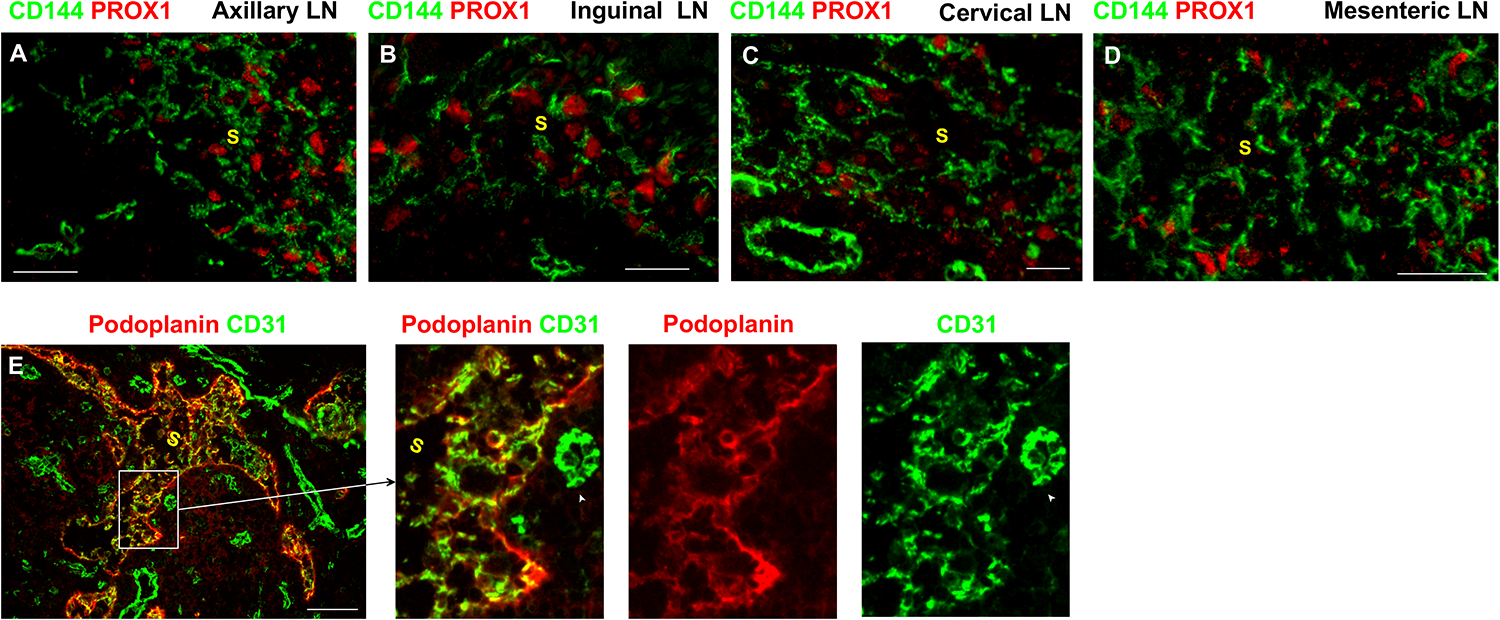
**

LECs in the sinuses consistently expressed PROX1 and CD144 in the axillary (A), inguinal (B), cervical (C) and mesenteric (D) LNs. LECs also expressed CD31 and podoplanin, while CD31^+^ blood vessels (arrowhead) lacked podoplanin expression (E). S, sinus. Scale bars represent 25µm (A-D) and 100 µm (E).

**
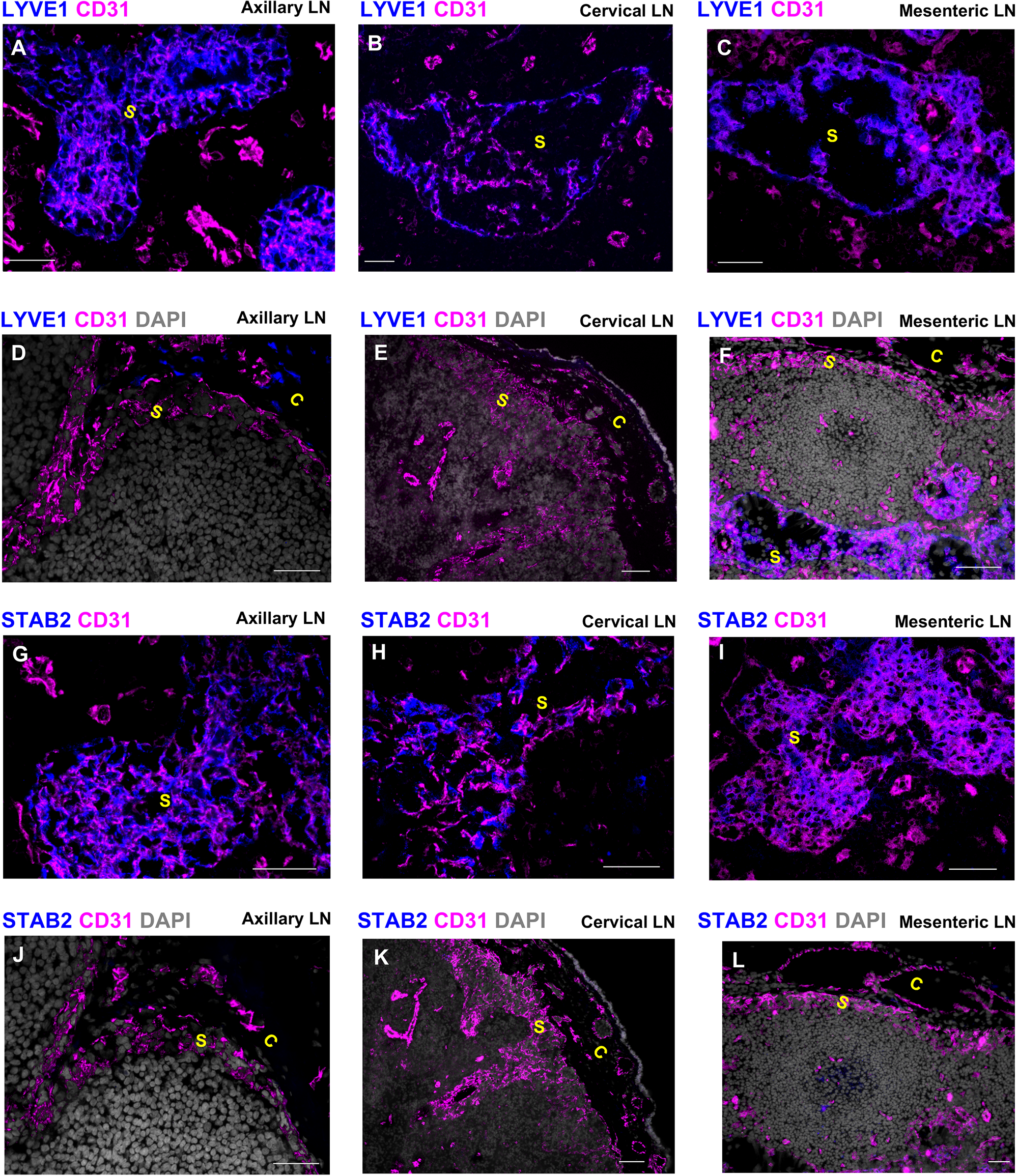
Figure S2.**

Expression of LYVE1 and STAB2 was examined in the axillary, cervical and mesenteric LNs. In these LNs, CD31^+^ LECs of the paracortical and medullary sinuses expressed LYVE1 (A-C) and STAB2 (G-I), whereas the LECs in the subcapsular and trabecular sinuses were negative for these markers (D-F, J-L). Grey represents DAPI staining of cell nuclei (D-F, J-L). C, capsule; S, sinus. All scale bars represent 50µm except in E, F and K (100µm).

**Figure S3.**

**
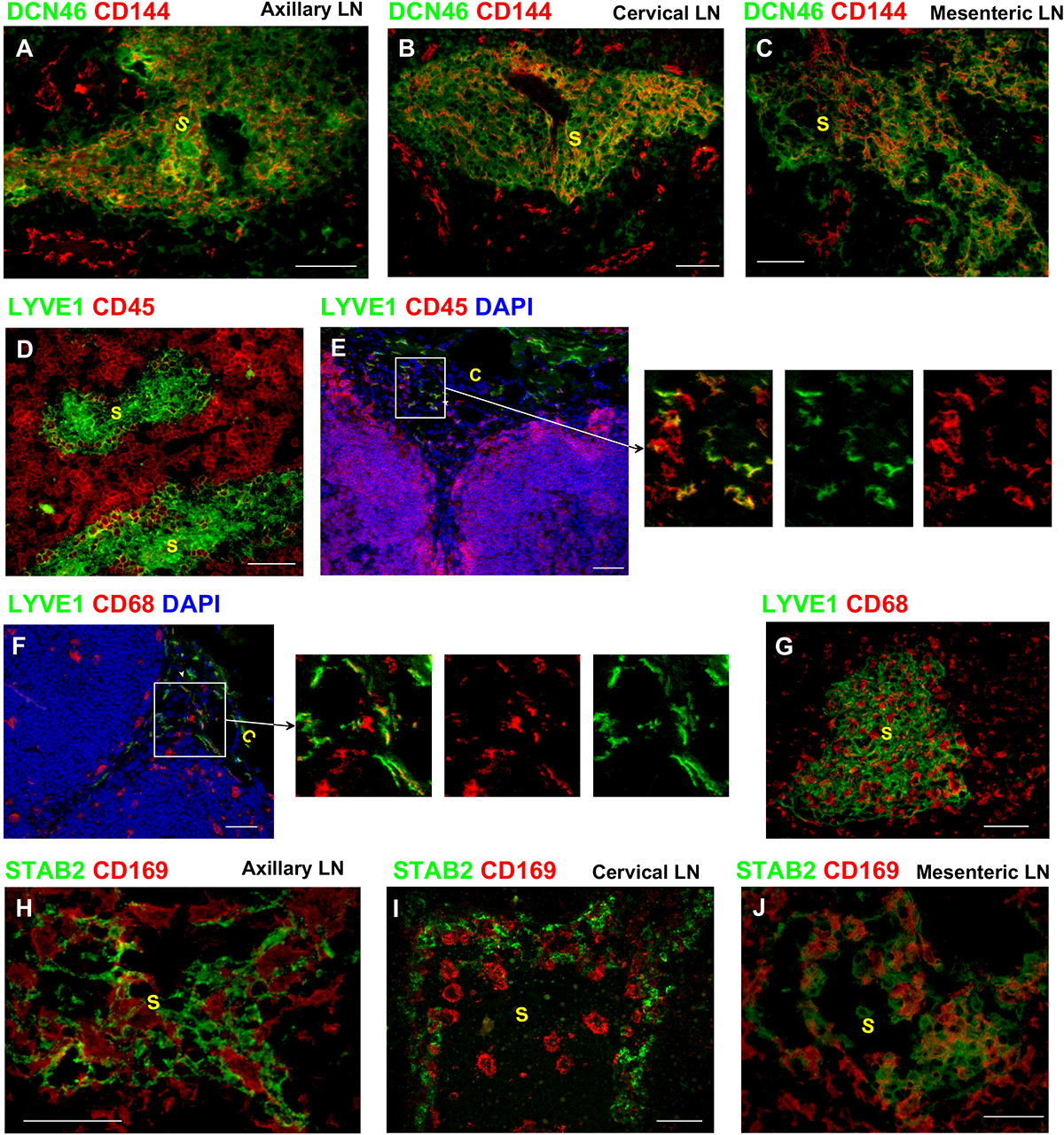
**

In the axillary, cervical and mesenteric LNs, CD144^+^ paracortical and medullary sinuses were densely packed with DCN46^+^ cells (A-C). The LYVE1^+^ LECs in the sinuses lacked expression of CD45 and CD68 (D, G), whilst the LYVE1^+^ cells in the capsule expressed these markers (E, F). CD169^+^ STAB2^-^ sinus APCs were intermingled with the CD169^-^ STAB2^+^ LECs in the axillary (H), cervical (I) and mesenteric (J) LNs. C, capsule; S, sinus.  All scale bars represent 50µm.

**Figure S4.**

**
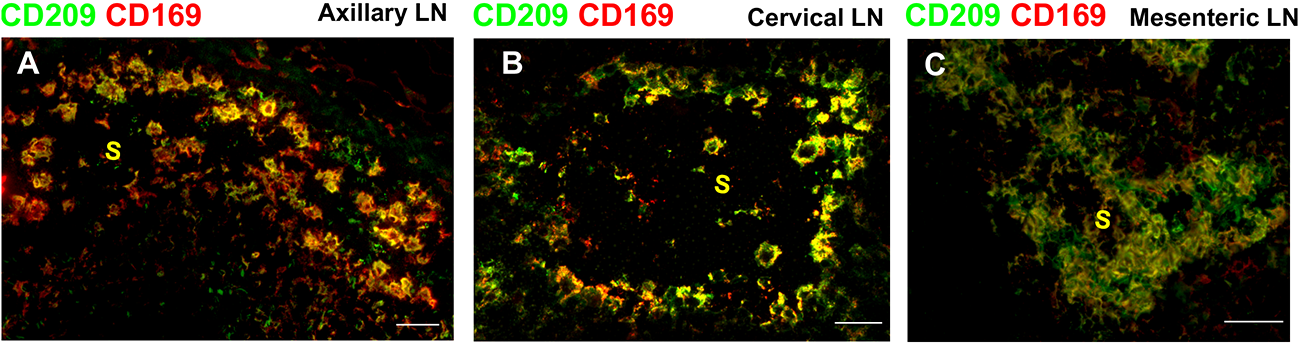
**

CD169^+^ sinus APCs expressed CD209 in the axillary (A), cervical (B) and mesenteric (C) LNs examined. S, sinus. All scale bars represent 50µm.
